# Personalized biosensors for point-of-care diagnostics: from bench to bedside applications

**DOI:** 10.7150/ntno.81485

**Published:** 2023-02-09

**Authors:** Pranjal Chandra

**Affiliations:** Laboratory of Bio-physio Sensors & Nanobioengineering, School of Biochemical Engineering, Indian Institute of Technology (BHU) Varanasi, Varanasi 221005, Uttar Pradesh, India

## Abstract

The most significant feature of translational point-of-care technology “Personalized biosensors” is that it can be done quickly and by clinical staff who are not trained in clinical laboratory sciences. Rapid test results can quickly give a doctor or other medical worker answers that can help them decide what to do or how to treat a patient. This is helpful almost everywhere, from the emergency room to a patient getting care at home. When a doctor meets a patient for the first time, during a flare-up of a known problem or when a new symptom shows up in a patient who is already being treated, having faster access to test results gives the doctor answers when they are with the patient or are about to see the patient which indicate the importance of point-of-care technologies and their future scope.

## Translational point-of-care diagnostics

The human body is a marvelous creation; it is a complicated creature of many different systems, each of which is trying to sustain the life we all too often take for granted. This is something that the human body does to keep us alive. In addition, all aspects of day-to-day life depend upon the biomedical sciences; this field of expertise is always developing as a consequence of the obligation to do so, and as a direct result, it has influenced the lives of a large number of people. It is not an exaggeration to claim that humans' life expectancy and longevity far surpass even the wildest dreams of people living even in our most recent history. It far exceeds even the wildest hopes of those living even in our most recent past. Concerns about people's and the public's health are among the most significant threats that humanity faces on a global scale. According to the World Health Organization, millions of people get sick with some form of the disease every year, and more than half of those people get very sick 1. Also, these harsh conditions have led to deadly progression. Statistical analyses done by both public and private groups have shown that wrong diagnoses cost the patient more and make it more likely that the condition will get worse. In a study done by a group from Duke University, the relationship between how a disease progresses and how bad it is, how much it hurts, and how much the patient suffers has been summed up 2. The progression of the disease after an infection increases exponentially. They have also taken the studies all the way down to the molecular level and explained that the symptoms of the disease show up a long time after the infection. In traditional settings, sick people are usually diagnosed with high-tech instruments that are usually found in centralized labs. Most of the time, protocols based on molecular and microbiology are used. So, by the time the clinical diagnosis is made, the patient's condition is already worsened. These strategies are robust and accurate, but they usually take a long time to implement, making them ineffective in emergencies, ambulatory, and remote situations. To get around these problems, a significant work has been done into ensuring that there are ways to diagnose. Importantly, finding the metabolic shift of the symptomless transition stage is an essential parameter of fighting any disease or disorder because a small clinical immune boost can cure the disease at this stage, which is critical to treat in the early stages. In the early stages, biomarkers are usually found in much lower concentrations, limiting the use of major conventional techniques and diagnostic devices and making room for various ultra-sensitive modules. Biosensors are one of the most important ways to find things at deficient levels 3-8.

Nanobiotechnology based interventional approaches have changed many aspects of basic and applied science in the last few centuries 9-11. On the other hand, nanobiotechnology has established a right junction between biomedical scientists, engineers, medical doctors, and society to develop cost effective nano-diagnostics probes which is our main area of research 12-16. Nano-diagnostics has significant contribution in the medical field, and now bio entrepreneurship is growing well in both developed and developing counties. Nowadays, the community is paying more attention to the huge demand of translational research and developing cost effective diagnostics probes/ devices to overcome the major limitations of conventional diagnosis methods. In particular, nano-diagnostics are usually better than lab-based techniques because they need less quantity of a sample, take less time to analyze, can detect problems onsite, and are more reliable which are essential parameters for developing clinically relevant diagnostics probes 3,5,17,18. In sensing, the first modules "biosensors" was made by Clark and Lyon in 1962 19 to detect glucose by oxidase enzyme electrode. Later in 1975, yellow spring instruments sold a version of this biosensor called the "Model 23A YSI analyzer." This was 10 years after this biosensor was first made. Researchers and manufacturers were inspired to start several new businesses by how well this module did in the marketplace. The first microbial biosensor was made in 1975, the first bedside artificial pancreas was made in 1976, and the first plasmon resonance immunosensor was made in 1984, among other essential biosensing mechanisms. In 1987, MediSense ExacTech Inc. developed a biosensor for blood glucose. After these path-setting products, several healthcare companies came out with i-STAT, Glucocard, and other products. In recent years, biosensors have been able to deal with a broader range of clinical problems and make more accurate, selective detections 18,20-23.

**Figure 1:**
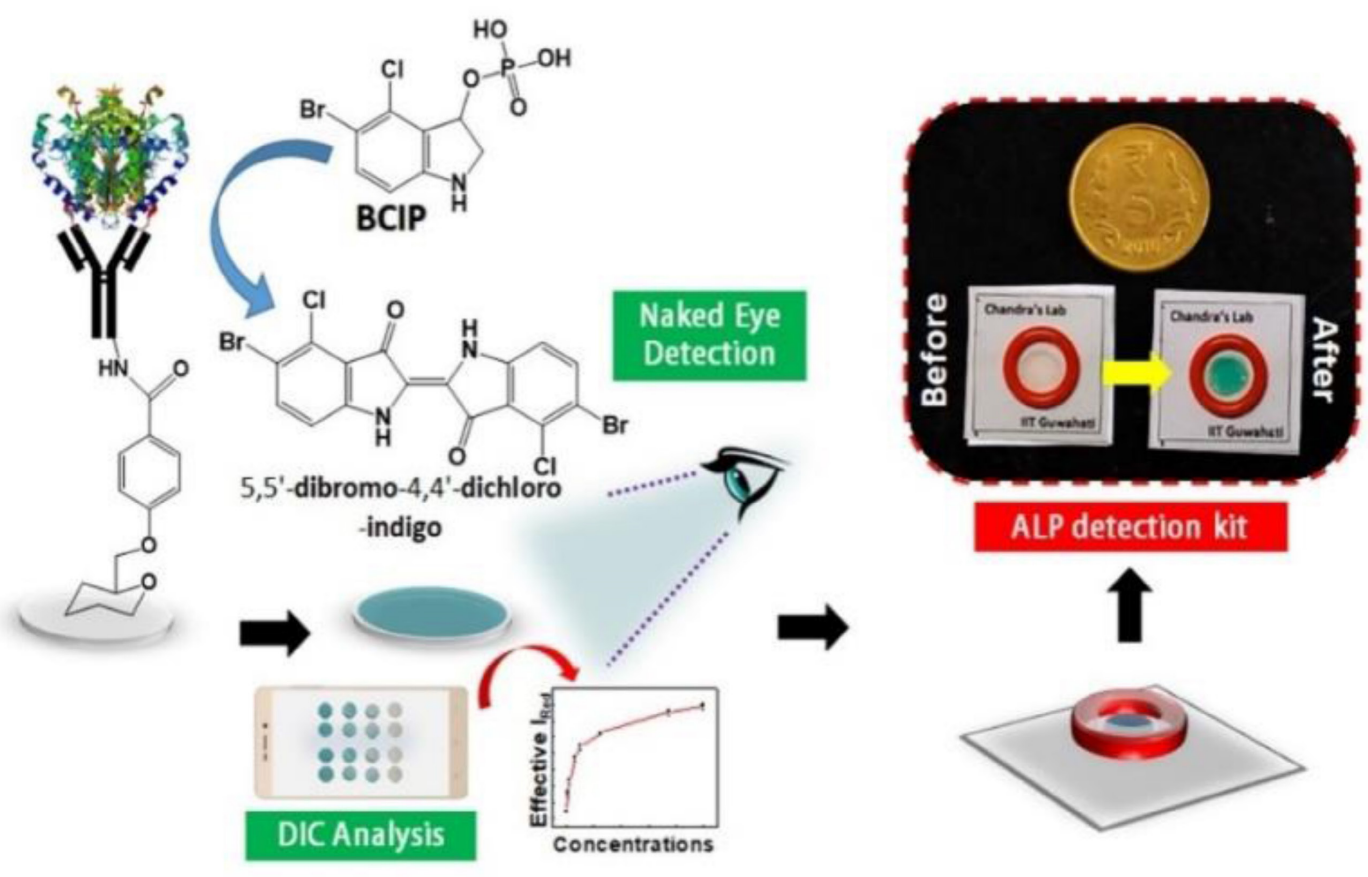
Schematic representation of the point-of-care ALP biosensor fabrication and detection principle (Reused with permission from 24).

Clinical diagnostics need equitable and reliable biosensors right away to deal with several clinical emergencies. After looking at the trends in health status worldwide, statisticians have predicted an alarming situation. There is always challenge for biomedical researchers, medical doctors, healthcare workers, the planning commission, and regulatory bodies to set the cost-effective approaches of nano diagnostics/sensors. Many reviews and research articles highlight several ways to prevent critical issues of biosensors to demonstrate accurate diagnoses. These methods can find biomarkers even before a disease has any symptoms. Biosensing prototypes have also been discussed in terms of how nanomaterials affect their sensing/or diagnosis abilities. Deployment of a dual sensor system (optical and electronic sensors) was a breakthrough for ultra-fast detection of alkaline phosphatase (ALP) in milk 24. For a common person, milk has a richness of almost all types of essential nutrients, *viz.,* carbohydrates, vitamins, proteins, fats, minerals, and calcium. Due to its immense nutritional value and health-related benefits, its commercialization has rapidly grown globally. Various nutrients make it prone to certain microbial invasions, which could harbor the potential causative agents of milk-borne infections. Thus, the milk is sterilized by heating and rapid instant cooling (pasteurization process) to check the microbial invasions for safer consumption. ALP content has been reported as an indicator molecule to ensure this pasteurization process for health benefits. Several methods have been reported for ALP determination in milk; however, their requirements of dedicated infrastructure, high end instruments, requirement of trained personnel and execution time limit their utility for onsite detection, which eventually costs a massive wastage of the raw milk during the processes 25. Moreover, the earlier ALP detection methods were unable to detect it quantitatively in miniaturized settings. To overcome these aforementioned issues, we have developed a smartphone-enabled*,* hand-held colorimetric optoelectronic device to detect ALP in a personalized setting **(Figure 1)**. The system consisting of a set of biosensors for detecting ALP in milk samples for comprehensive screening, including a paper-based and bioelectronic chip-based module. The paper-based module can detect ALP in a couple of minutes to screen the raw milk and human serum samples. Assisted by a smartphone, ALP has been quantified, where a higher concentration infers the milk is from a diseased /udder-infected cow.

In the second module, we introduced a sensitive bio-electronic chip-based platform for efficiently discriminating raw and pasteurized milk from synthetic milk. Recently, we have also integrated this system with Raspberry Pi to develop a hand-held palm-sized optical sensing device named *"OPTILIZER"* for ALP detection in miniaturized settings which takes merely 30 seconds of sensing as compared to the conventional diagnostic approaches. Our developed translational technology “bioelectronic device” is highlighted on YouTube link^1^ which was successfully tested for ALP detection in milk and human serum samples and a patent.^2^

**Figure 2:**
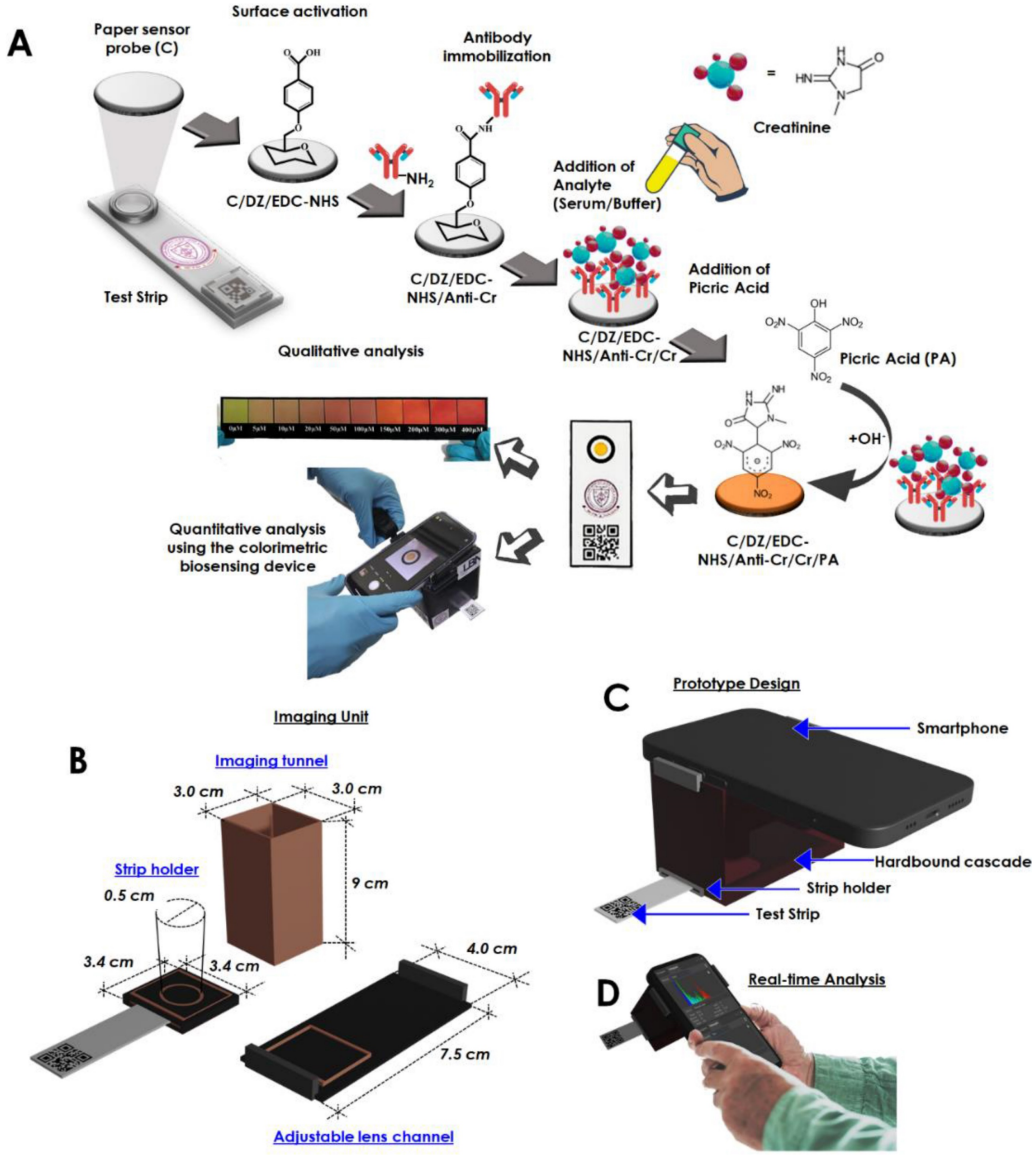
Schematic representation of the fabrication of the creatinine sensing device. (A) Illustration of the step-by-step fabrication of the sensor probe and the proposed colorimetric sensing device, (B) Specified dimension of the internalized segments of the device construction, optimized for image capturing and processing, (C) Graphical impression of the developed prototyped device, (D) Real time analysis and image processing through the developed system obtaining reading for creatinine detection (Reused from 26, the work is licensed under a Creative Commons Attribution 4.0 International License)

In another paper, we talked about an optical sensing device that makes it easy to find creatinine at the point of care 26. The biosensor that was made could measure how much creatinine was in the blood by looking at how the color changed. The sensor has been added to a smartphone to make a palm-sized device that can analyzed creatinine in a person's own environment **(Figure 2)**. The creatinine-selective antibody was attached to a sensing probe through a few easy steps of chemical modification **(Figure 2A)**. The reaction between picric acid and creatinine is used to do the quantitative analysis. The detailed internal configuration of the sensor device has been depicted in **Figure 2B**. **Figure 2C** and **D** shows the graphical illustration and the real-time sensing analysis practice of the device prototype. This reaction was picked up by the antibody-functionalized sensor probe. The changes in color intensity and creatinine concentrations exhibit an outstanding dose-dependent correlation in two separate dynamic ranges, from 5 to 20 μM and 35 to 400 μM. The detection limit for this correlation is 15.37 (±0.79) nM, and it is present in both of these ranges. Using the biosensor, several molecules that have the potential to cause issues were examined, including albumin, glucose, ascorbic acid, citric acid, glycine, uric acid, Na+, K+, and Cl^-^, however, the biosensor did not detect any cross-reactivity between any of these substances. It was determined that the system was able to assess the amount of creatinine even in serum samples that had been tampered with, and the percentage recoveries found ranged from 89.71 to 97.30 %. It was discovered that the newly created biosensor is stable for a period of up to 28.

## Point-of-care technology in clinical practice: from bench to bedside

Point-of-care diagnostics is now leaning heavily in the direction of smart devices outfitted with mobile healthcare, which has the potential to completely transform the monitoring and administration of customized healthcare, paving the way for the next generation of point-of-care technologies. The most promising point-of-care technologies have already been developed for the readout of colorimetric, lateral flow, electrochemical, chemiluminescent, fluorescent, and label-free tests; detection of cells, biomolecules, nanoparticles, and microbes; and other diagnostic applications. A wide variety of mobile healthcare technologies have already been developed. The number of people who use cell phones has now surpassed 7.4 billion, with 70 percent of those users being in developing nations, with an urgent need for point-of-care diagnostics. To monitor and manage fundamental health parameters, such as blood glucose levels, blood pressure, weight, body analysis, pulse rate, electrocardiogram, and physical activity, a number of devices based on mobile phones and intelligent applications have been brought to market. However, protecting personal information and privacy is a significant worry for mobile healthcare. This is in addition to the need of developing worldwide cloud-computing standards and the administration of "Big Data." In the future years, these difficulties will be properly addressed thanks to the worldwide efforts that are being made. In addition, the development of complementing technologies may eventually result in the creation of next-generation point-of-care diagnostics. The day is not far off when most people will utilize point-of-care diagnostics, enabling individuals to keep an eye on and take responsibility for their own health management.

## Major obstacles for developing point-of-care technologies

- There is a possibility that label-free assays could provide quantitative real-time measurements. When it comes to dealing with analytes in intricate matrices, however, they are afflicted with a high level of non-specific binds and aberrant findings.
- The timely identification and beginning of targeted antimicrobial therapy utilizing biosensor technologies that are portable, sensitive, specific, and cost-effective are essential to clinical care in settings that lack centralized administration and have limited access to resources. This is because these technologies are required for the timely identification of pathogens and the beginning of targeted antimicrobial therapy.
- Biosensor technologies have a tremendous challenge in the shape of the matrix effects of clinical samples; hence, it is unusual to come across biosensors that display excellent performance when used with actual clinical samples.
- The practice of *in vitro* diagnostics, as it is now carried out, requires the utilization of a sophisticated laboratory, personnel with required skills, as well as expensive and heavy equipment.
- Nevertheless, increasing the number of binding events and amplification tags in labelled assays might improve specificity and sensitivity. On the other hand, multistep procedures might make the experiment more difficult to understand.
- The development of chip-based sample preparation processes in microfluidic systems is an absolutely necessary step in order to get biosensors out of the laboratory and into clinical settings. These procedures ought to eliminate matrix inhibitors, enrich target analytes, and lessen the volume of the sample.
- The systems management of major modules of a biosensor, which are a detection mechanism, microfluidics-based preparative strategies, and a transducer, into a completely automated and standalone platform is, and will continue to be, the most critical challenge for the commercial exploitation of point-of-care devices. These major modules include a detection mechanism, a microfluidics-based preparative strategy, and a transducer.

## Conclusion and perspective

In this article, we discussed the road map for the development of individualized biosensors for use in point-of-care diagnostics, from the laboratory to the patient's bedside. The editorial highlighted several cases of point-of-care diagnostics that have demonstrated clinical utility. Though microfluidics technology has shown promise for improving point-of-care diagnostics for decades, it is unlikely that an ultrasensitive lab-on-a-chip device that can directly handle raw materials in a tailored setting will be commercially accessible within the next five years. When calculating the genuine clinical advantages of the test, as measured by disability-adjusted life years, the cost-effectiveness of the technique is the primary motivator for its commercialization. One technological approach to making the most of the benefits on offer is to create a general integrated system that can process a wider range of clinical samples, including urine, blood, and saliva, for different infectious viruses or pathogens. The bulk of researchers in the fields of microfluidics do not have access to raw samples, and they have little experience with marketable devices, which creates a gap between academic ideas and clinical validation. Future research institutions, hospitals, and businesses will need to work together more closely to make lab-on-a-chip technology a reality. I think it's crucial for young people from these various groups to remember that they need to bridge their expertise and tackle the problems in a more positive way.

## Footnotes

1
https://youtu.be/SlNHkYYQtvQ

2 (Patent Application No.: 202111046586: 2021-10-12)
